# Obliteration of a glycinergic projection to the medial geniculate in an animal model of autism

**DOI:** 10.3389/fncel.2024.1465255

**Published:** 2024-10-17

**Authors:** Yusra Mansour, Randy Kulesza

**Affiliations:** ^1^Department of Otolaryngology—Head and Neck Surgery, Detroit, MI, United States; ^2^Department of Anatomy, Lake Erie College of Osteopathic Medicine, Erie, PA, United States

**Keywords:** hearing, brainstem, autism, development, neurodevelopmental disorders

## Abstract

Auditory dysfunction affects the vast majority of people with autism spectrum disorder (ASD) and can range from deafness to hypersensitivity. *In utero* exposure to the antiepileptic valproic acid (VPA) is associated with significant risk of an ASD diagnosis in humans and timed *in utero* exposure to VPA is utilized as an animal model of ASD. VPA-exposed rats have significantly fewer neurons in their auditory brainstem, thalamus and cortex, reduced ascending projections to the midbrain and thalamus and reduced descending projections from the cortex to the auditory midbrain. Consistent with these anatomical changes, VPA-exposed animals also have abnormal auditory brainstem responses. We have recently described a significant ascending projection from calbindin-positive neurons in the medial nucleus of the trapezoid body (MNTB) to the ventral division of the medial geniculate (vMG) in rats that bypasses the central nucleus of the inferior colliculus (CNIC). Since we found that axonal projections to the vMG in VPA-exposed rats are reduced beyond what is predicted from neuron loss alone, we hypothesize that VPA exposure would result in a significant reduction in the MNTB projection to the vMG. We examined this hypothesis by quantifying the proportion of retrogradely-labeled neurons in the MNTB of control and VPA-exposed animals after injections of retrograde tracers in the CNIC and vMG in control and VPA-exposed animals. Our results indicate that in control animals, the MNTB forms the largest projection from the superior olivary complex to the MG and that this projection is nearly abolished by *in utero* VPA exposure.

## Introduction

Autism spectrum disorder (ASD) is a developmental disability characterized by social, communication and behavioral difficulties (Allen, 1988; Wing, 1997; American Psychiatric Association, 2013; CDC.gov, 2024). Approximately one in 36 children will be diagnosed with ASD and this is four times more common in males (CDC.gov, 2024). There are several key signs and symptoms of ASD, but the vast majority of subjects have some degree of auditory dysfunction (Greenspan and Wieder, 1997; Tomchek and Dunn, 2007; Bolton et al., 2012; reviewed in Mansour et al., 2021a) and this can range across individuals from deafness to hypersensitivity to sounds (Roper et al., 2003; Alcántara et al., 2004; Khalfa et al., 2001; Szelag et al., 2004; Teder-Sälejärvi et al., 2005; Gravel et al., 2006; Tharpe et al., 2006; Russo et al., 2009). Indeed, many individuals with ASD have longer latency auditory brainstem responses (ABR) (Ornitz, 1969; Student and Sohmer, 1978; Rosenblum et al., 1980; Sohmer, 1982; Tanguay et al., 1982; Gillberg et al., 1983; Sersen et al., 1990; Thivierge et al., 1990; Wong and Wong, 1991; Maziade et al., 2000; Kwon et al., 2007; Roth et al., 2012; Azouz et al., 2014; Tas et al., 2007; Miron et al., 2018; Ramezani et al., 2019; Delgado et al., 2023). Consistent with hearing impairments from a developmental etiology, we have identified significant and consistent auditory brainstem hypoplasia in the brainstem of individuals with ASD (Kulesza and Mangunay, 2008; Kulesza et al., 2011; Lukose et al., 2015; Mansour and Kulesza, 2020). Specifically, in our study of the superior olivary complex (SOC) in a cohort of 28 subjects with ASD ranging from 4 to 39 years of age, we found significantly fewer neurons and surviving neurons were significantly smaller across nearly all constituent nuclei, including the medial nucleus of the trapezoid body (MNTB). In addition, several of these subjects had marked gliosis in and around the medial superior olive (MSO) and/or islands of ectopic neurons posterior and lateral to the SOC (Lukose et al., 2015).

*In utero* exposure to the antiepileptic drug valproic acid (VPA) is associated with elevated risk of an ASD diagnosis in humans (Moore et al., 2000; Williams et al., 2001; Rasalam et al., 2005; Koren et al., 2006; Bromley et al., 2013; Christensen et al., 2013; Hernández-Díaz et al., 2024; Pack et al., 2024). Accordingly, timed *in utero* exposure to VPA is a biologically relevant and validated animal model of ASD (rodents: Rodier et al., 1996; Mabunga et al., 2015; primates: Zhao et al., 2019). Consistent with the neuropathological changes we identified in the SOC of human subjects with ASD, animals exposed to VPA *in utero* have significantly fewer neurons in the ventral cochlear nuclei (VCN), SOC, nuclei of the lateral lemniscus (NLL), central nucleus of the inferior colliculus (CNIC), and medial geniculate (MG; Lukose et al., 2011; Zimmerman et al., 2018; Mansour et al., 2019). VPA-exposed animals also have fewer neurons across all layers of the auditory cortex with smaller pyramidal and non-pyramidal neurons in auditory association areas (Kosmer and Kulesza, 2024). Besides having fewer neurons throughout the auditory brainstem and forebrain, VPA-exposed animals have reduced ascending projections to the CNIC (Zimmerman et al., 2020) and MG (Mansour et al., 2021b) and reduced descending projections from layer VI of auditory cortex to the CNIC (Kosmer and Kulesza, 2024). VPA-exposed animals have fewer calbindin (CB) immunoreactive neurons in several locations, including the octopus cell area in the VCN, MNTB (Zimmerman et al., 2018), dorsal nucleus of the lateral lemniscus (DNLL; Mansour et al., 2019), primary auditory cortex (Kosmer and Kulesza, 2024), and cerebellum (Main and Kulesza, 2017) and fewer CB+ puncta in vestibular nuclei (Mansour et al., 2022). VPA-exposed animals have significantly more cFOS+ neurons in the VCN, MNTB and CNIC after exposure to pure tone stimuli, consistent with disruption of inhibitory circuits (Dubiel and Kulesza, 2016). Finally, VPA-exposed animals have abnormal auditory brainstem responses, including elevated thresholds, and longer latency responses for wave III, IV and V, consistent with auditory brainstem dysfunction (Malhotra and Kulesza, 2023).

**Figure 1:**
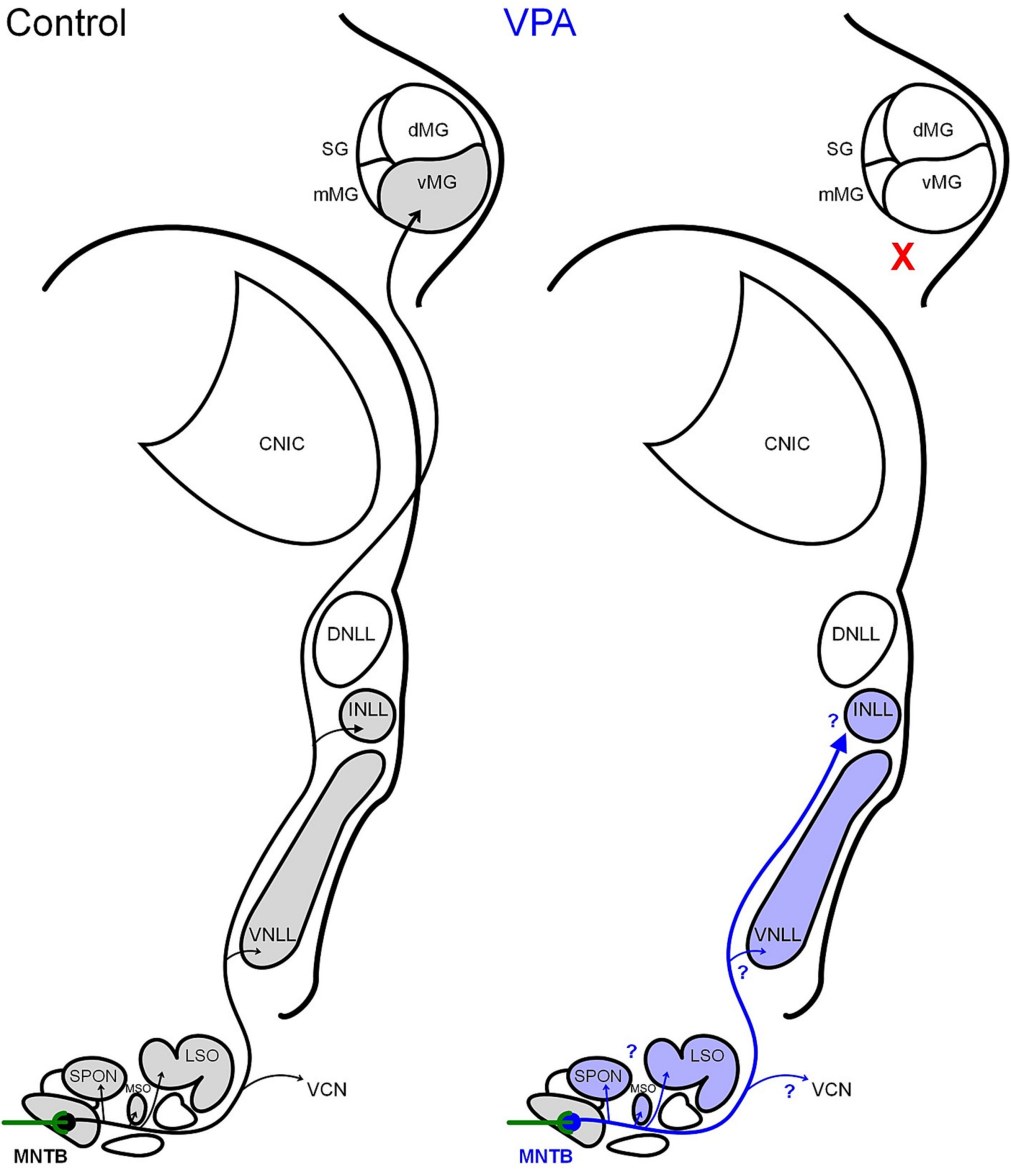
Schematic of MNTB projections. In control animals, the MNTB receives its main input from the contralateral VCN via the calyx of Held and projects within the SOC, to the ipsilateral VCN, to the VNLL, INLL, and MG. Our tract tracing results indicate that VPA exposure abolishes the MNTB projection to the MG. Currently, it is unclear if VPA exposure impacts other projections from the MNTB.

The rat MNTB is composed primarily of glycinergic, CB+ principal neurons that receive input from globular bushy cells (GBCs) in the contralateral VCN via the calyx of Held (Morest, 1968a,b; Ottersen and Storm-Mathisen, 1984; Friauf and Ostwald, 1988; Arai et al., 1991; Résibois and Rogers, 1992; Lohmann and Friauf, 1996; Smith et al., 1991). MNTB principal neurons project within the ipsilateral SOC to the medial and lateral superior olives (MSO and LSO, respectively; Spangler et al., 1985; Helfert et al., 1989), and the superior paraolivary nucleus (SPON; Kuwabara et al., 1991; Banks and Smith, 1992; Sommer et al., 1993; Kulesza, 2007; see Figure 1, control). The MNTB also sends a descending projection to the GBC area of the ipsilateral VCN (Schofield, 1994) and ascending projections to both the ventral and intermediate nuclei of the lateral lemniscus (VNLL, INLL; Spangler et al., 1985; Sommer et al., 1993; Smith et al., 1998; Kelly et al., 2009; Saldaña et al., 2009; see Figure 1, control). Injections of retrograde tracers into nuclei further rostral in the brainstem such as the DNLL and CNIC indicate that only rare MNTB neurons project to these targets (rat: Beyerl, 1978; Druga and Syka, 1984; Coleman and Clerici, 1987; Kelly et al., 1998, 2009; Saldaña et al., 2009; guinea pig: Schofield and Cant, 1992; cat: Adams, 1979; Brunso-Bechtold et al., 1981; gerbil: Nordeen et al., 1983; Cant and Benson, 2006; mole: Kudo et al., 1990). However, we have recently demonstrated a projection from the MNTB to the ipsilateral MG that bypasses the CNIC in Sprague–Dawley rats (Burchell et al., 2022). Specifically, approximately 40% of MNTB neurons project to the ipsilateral ventral division of the MG (vMG; Burchell et al., 2022). Because VPA exposure results in significantly reduced ascending projections from the SOC to the MG, we hypothesized that *in utero* VPA exposure will result in a significant reduction in this ascending glycinergic projection from the MNTB to the MG. We examined this hypothesis in a library of retrograde tracer injections into the CNIC and MG from control and VPA-exposed animals (Zimmerman et al., 2020; Mansour et al., 2021b).

## Methods

**Figure 2:**
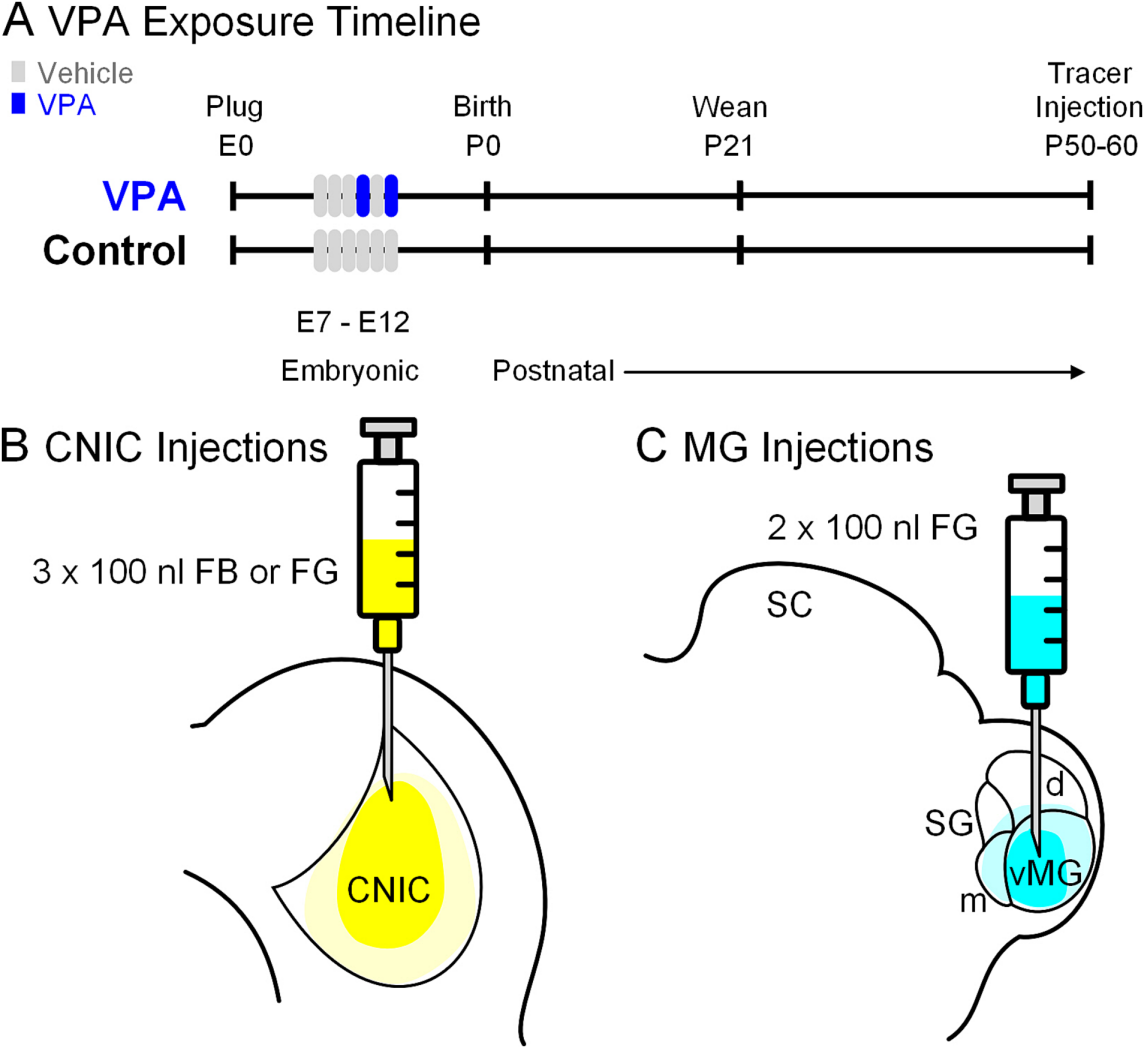
Experimental paradigm. Panel **(A)** shows the timing of VPA exposure, weaning and tracer injections. The experimental timeline (embryonic day 0; E0) started with identification of a vaginal plug. Pregnant females in the control group were fed peanut butter (vehicle) from E7 through E12; those in the experimental group received peanut butter on E7-9 and E11 and peanut butter + VPA on E10 and E12. Pups were weaned on P21 and stereotaxic injections of retrograde tracers were made between P50-60. Three injections (100 nL each) were made into the CNIC **(B)**. In a separate group of animals, two injections (100 nL each) were made into the MG **(C)**.

All handling and surgical procedures were approved by the LECOM Institutional Animal Care and Use Committee (protocols #16-02, 19–04, 20–02 & 21–03) and conducted in accordance with the National Institute of Health Guide for the Care and Use of Laboratory Animals. Sprague–Dawley rats were maintained on a 12 h light/dark cycle with *ad libitum* access to food and water. *In utero* exposure to VPA was performed per our previous work in this model (Figure 2A; Main and Kulesza, 2017; Zimmerman et al., 2018; Mansour et al., 2019; Zimmerman et al., 2020; Mansour et al., 2021b; Mansour and Kulesza, 2021; Mansour et al., 2022; Malhotra and Kulesza, 2023; Kosmer and Kulesza, 2024). All dams were fed 3.1 g of peanut butter on embryonic days (E) 7–12. Dams in the VPA group were fed peanut butter mixed with 800 mg/kg of VPA on E10 and E12 (Figure 2A). Both control and VPA-exposed dams were permitted to deliver pups without interference and pups were weaned on postnatal day (P) 21. Only male pups were included in the study because gender-specific effects of VPA exposure are established (Schneider et al., 2008). We conducted this study under the assumption that all male pups in a given litter were equally impacted by VPA exposure; our previous studies provide data consistent with this strategy (Main and Kulesza, 2017; Zimmerman et al., 2018; Mansour et al., 2019; Zimmerman et al., 2020; Mansour et al., 2021b; Mansour and Kulesza, 2021).

Animals receiving tracer injections were anesthetized with vaporized isoflurane (5% isoflurane in oxygen for induction, 2–3% for maintenance at 1.2 L/min). When animals were unresponsive to toe pinch, they were fitted with an anesthesia mask and secured in a stereotaxic frame with non-rupture ear bars. Body temperature was maintained with an electric heating pad. The animal’s scalp was cleaned with iodine solution and injected with 0.25% bupivacaine; eyes were covered with ophthalmic ointment or closed and covered over with the anesthesia mask. A midline incision was made over the parietal and occipital bones and the dorsal aspect of the brain was approached via stereotaxic craniotomy. All injections were made with a tracer-dedicated 1 μL Hamilton KH Neuros syringe (32-gauge, four points).

We studied projections from the MNTB to the CNIC in injection site-matched cases from seven control and eight VPA-exposed animals (Figure 2B). In all animals, the CNIC was approached 0.2 mm rostral to lambda, 1.5 mm to the right of the midline. A depth measurement was taken from the surface of the dura mater and deposits of 100 nL of Fast Blue (FB; 2.5% in water; Polysciences) or Fluorogold (FG, 4% in saline; Fluochrome) were made at depths of −3.6, − 3.2, and − 2.6 mm for a total injected volume of 300 nL.

We studied projections from the MNTB to the MG in injection site-matched cases from six control and six VPA-exposed animals (Figure 2C). The stereotaxic coordinates were the same for all control and VPA-exposed animals: 5.6 mm caudal to bregma and 3.4 mm to the right of the midline (as indicated by Paxinos and Watson, 2007). Injections of FG were made as described above. A depth measurement was taken from the surface of the dura mater and deposits of 100 nL of FG were made at depths of −5.8 and − 5.0 mm for a total injected volume of 200 nL.

The syringe was left in place for 10 min after the final injection to permit diffusion of the tracer. After the syringe was removed, the scalp wound was injected with lidocaine and sutured. Animals were removed from anesthesia and placed in their home cage and monitored until they were able to stand on all fours. Six-days following the surgery, animals were anesthetized with isoflurane and perfused through the ascending aorta first with 0.9% saline and then 4% paraformaldehyde (PFA) in phosphate buffered saline (pH 7.4; “fixative”). Brains were dissected from the skull and the right side (ipsilateral to the tracer injection) was marked with a syringe needle and post-fixed for at least 24 h. Twenty-four hours before frozen sectioning, brains were transferred into cryoprotectant (30% sucrose in fixative). Brains were sectioned in the coronal plane at a thickness of 50 μm and sections were collected in PBS from the cochlear nucleus through the injection site in the CNIC or MG in three wells. Injection sites were recovered from well 3; sections from well 2 were counter stained with Neurotrace Red (NT; Invitrogen), mounted onto glass slides from cresyl gelatin, coverslipped with Entellan (Millipore Sigma) and photographed with an Olympus CKX41 microscope with epifluorescence and a DP71 camera. The rostrocaudal borders of the MNTB, SPON and dorsalmedial wedge (DMW) were as previously delineated (Kulesza et al., 2002; Mansour et al., 2021b; Burchell et al., 2022). For each section including the MNTB, two images were collected—one of NT (using a rhodamine filter cube) and one of FB/FG labeling (using a UV filter cube) using a 20 × objective. Each pair of images was combined using the z-stack feature in Fiji (Schindelin et al., 2012) to form a single layer image containing overlayed NT and FB/FG labeling.

Counts of FB/FG + MNTB neurons were made from 4 to 6 stacked images per animal. The overlayed FB/FG and NTR images were imported into Fiji and analyzed with the cell counting feature. In these images, neurons were considered FB or FG + if they had blue or yellow fluorescent labeling within a cell body contour. Neurons were considered negative if the cell body demonstrated NT labeling and lacked any blue/yellow fluorescence. At least 80 MNTB neurons were analyzed in each overlayed image and counts from these 4–6 images were averaged, resulting in a single percentage of FB/FG + MNTB neurons per animal. In Table 1, we re-examine previously published data from our study of ascending projections to the MG from the rat SOC (Mansour et al., 2021b) to demonstrate the relative size of the MNTB projection to the MG. Morphology of NTR and FB/FG-labeled MNTB neurons was quantified as previously described (Zimmerman et al., 2018).

**Table 1:** Neuron loss and projection changes in VPA-exposed animals.

	Number of neurons in nucleus^a^^,^^b^^,^^d^		MG neurons per total number of neurons in nucleus^a^^,^^b^^,^^c^			Total number of neurons projecting to MG^c^		MG neurons per projecting neurons in nucleus^c^			Projection Change^c^
	A	B	C	D	E	F	G	H	I	J	K
Calculation			46,796/A	23,403/B	D/C	A*%FG+	B*%FG+	46,796/F	23,403/G	I/H	E-J
	Control	VPA	Control	VPA	VPA/C	Control	VPA	Control	VPA	VPA/C	VPA/C
vMG + mMG	46,796	23,403									
						(36.7%)	(1.6%)				
IL MNTB	6,591	5,300	7.10	4.41	0.62	2,419	84.8	19.34	275.97	12.56	**−11.94**
IL LSO	2,586	1,935	18.10	12.09	0.67	173.26	75.47	270.09	310.09	1.15	−0.48
CL LSO	2,586	1,935	18.10	12.09	0.67	57.67	157.70	811.48	148.40	0.18	+0.49
IL MSO	1,201	517	38.96	45.27	1.16	148.56	38.93	314.99	601.15	1.91	−0.75
IL SPON	2,265	1,302	20.66	17.97	0.87	450.96	155.20	103.77	150.79	1.45	−0.58
IL DMW	2,407	1,803	19.44	12.98	0.67	883.37	485.91	52.97	48.16	0.91	−0.24
CL DMW	2,407	1,803	19.44	12.98	0.67	403.17	98.26	116.07	238.17	2.05	−1.38
IL VNTB	3,244	2,606	34.13	22.5	0.66	389.28	260.6	120.21	89.80	0.74	−0.08
CL VNTB	3,244	2,606	34.13	22.5	0.66	32.44	26.06	1,442.54	898.04	0.62	0.04
CL VCN	23,111	15,280	2.02	1.53	0.76	7,164.41	825.12	6.53	28.36	4.34	−3.59

a Zimmerman et al. (2018).

b Mansour et al. (2019).

c Mansour and Kulesza (2020).

d Mansour and Kulesza (2021).

GraphPad Prism (10.1, San Diego, CA) was used to generate descriptive statistics and conduct all statistical comparisons. Data that fit a normal distribution are presented in the text as mean ± standard deviation (SD); data that did not fit a normal distribution are presented as the median with the 95% confidence interval of the median. Specifically, the number of neurons projecting to the CNIC and MG were compared with Mann–Whitney (one-tailed) and neuronal morphology was compared with ANOVA with Tukey’s multiple comparison test. Differences were considered statistically significant if *p* values were < 0.05.

## Results

### Projections to the CNIC

**Figure 3:**
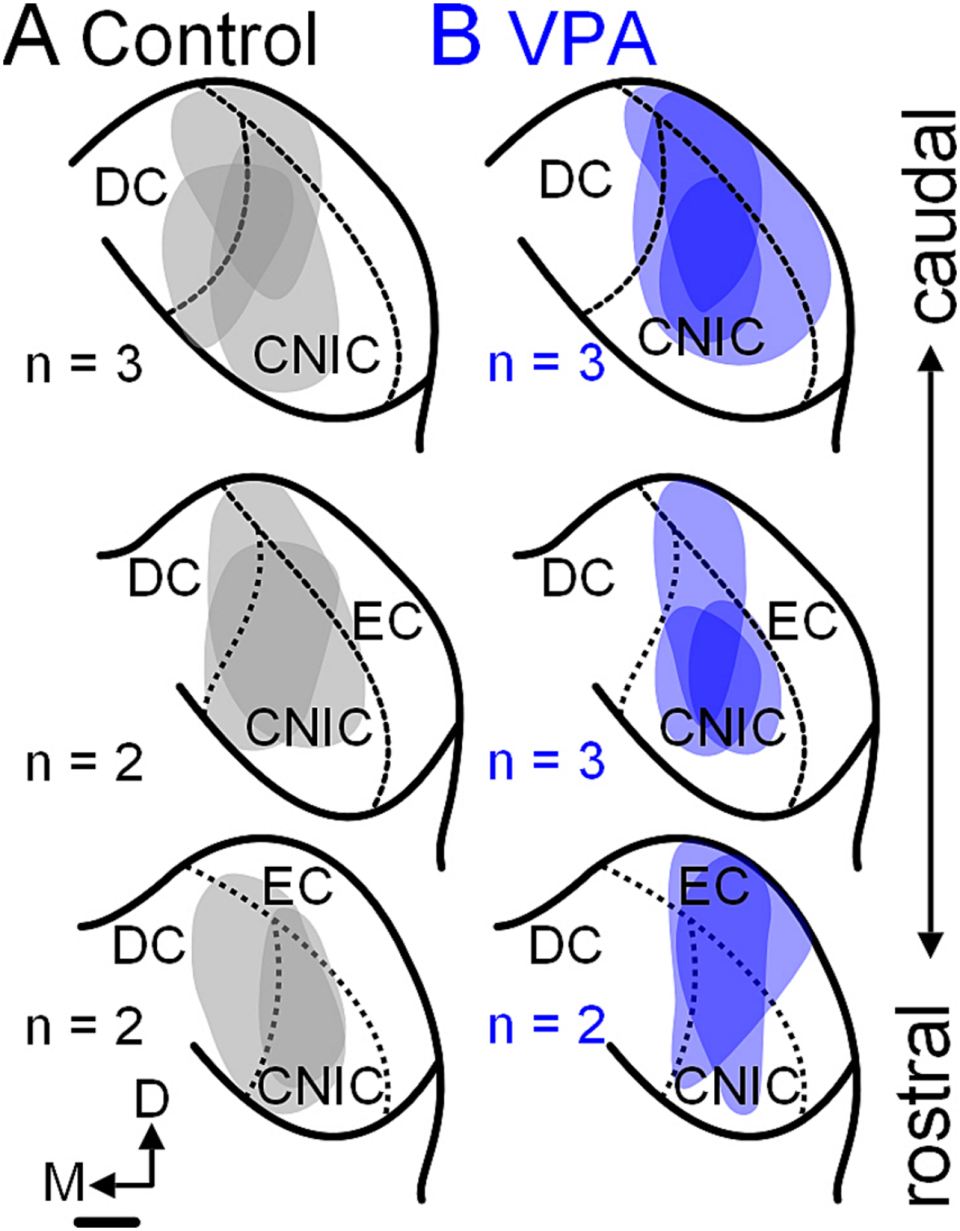
Tracer injection sites in the CNIC. Injection sites for the seven control animals are shown in panel **(A)** and those for the eight VPA-exposed animals are shown in panel **(B)**. These cases were selected to match injections site size and rostrocaudal distribution of injections between control and VPA-exposed animals. The scale bar is equal to 500 μm. D, Dorsal; DC, Dorsal cortex; EC, External cortex; and M, Medial.

**Figure 4:**
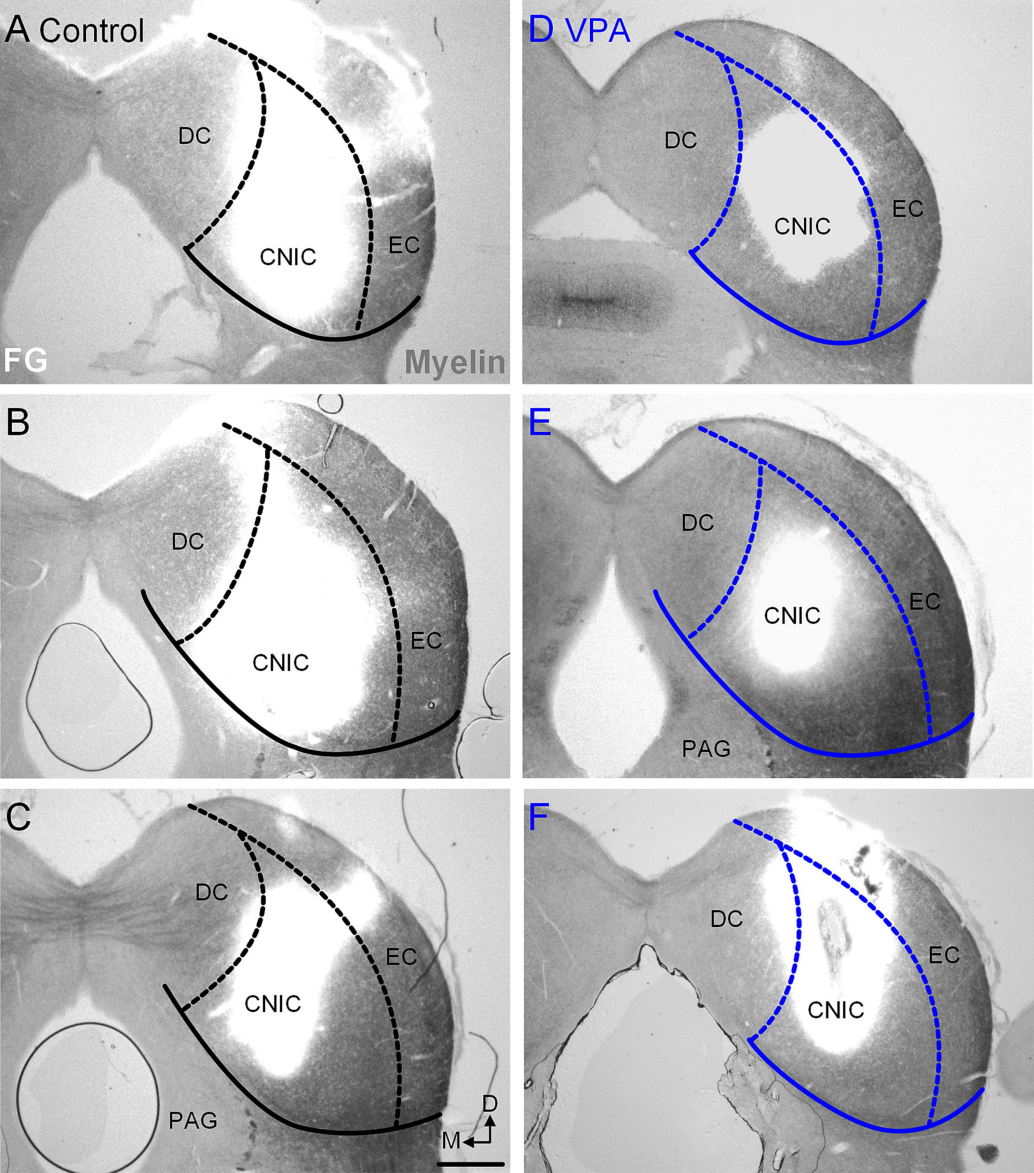
Injection sites in the CNIC. Representative examples of recovered injections sites in the CNIC are provided for control **(A–C)** and VPA-exposed animals **(D–F)**. The images were taken with simultaneous illumination with white light and a mercury lamp with a UV filter cube. Myeloarchitecture is shown in gray and the tracer injection in white. The scale bar is equal to 500 μm. D, Dorsal; DC, Dorsal cortex; EC, External cortex; M, Medial.

**Figure 5:**
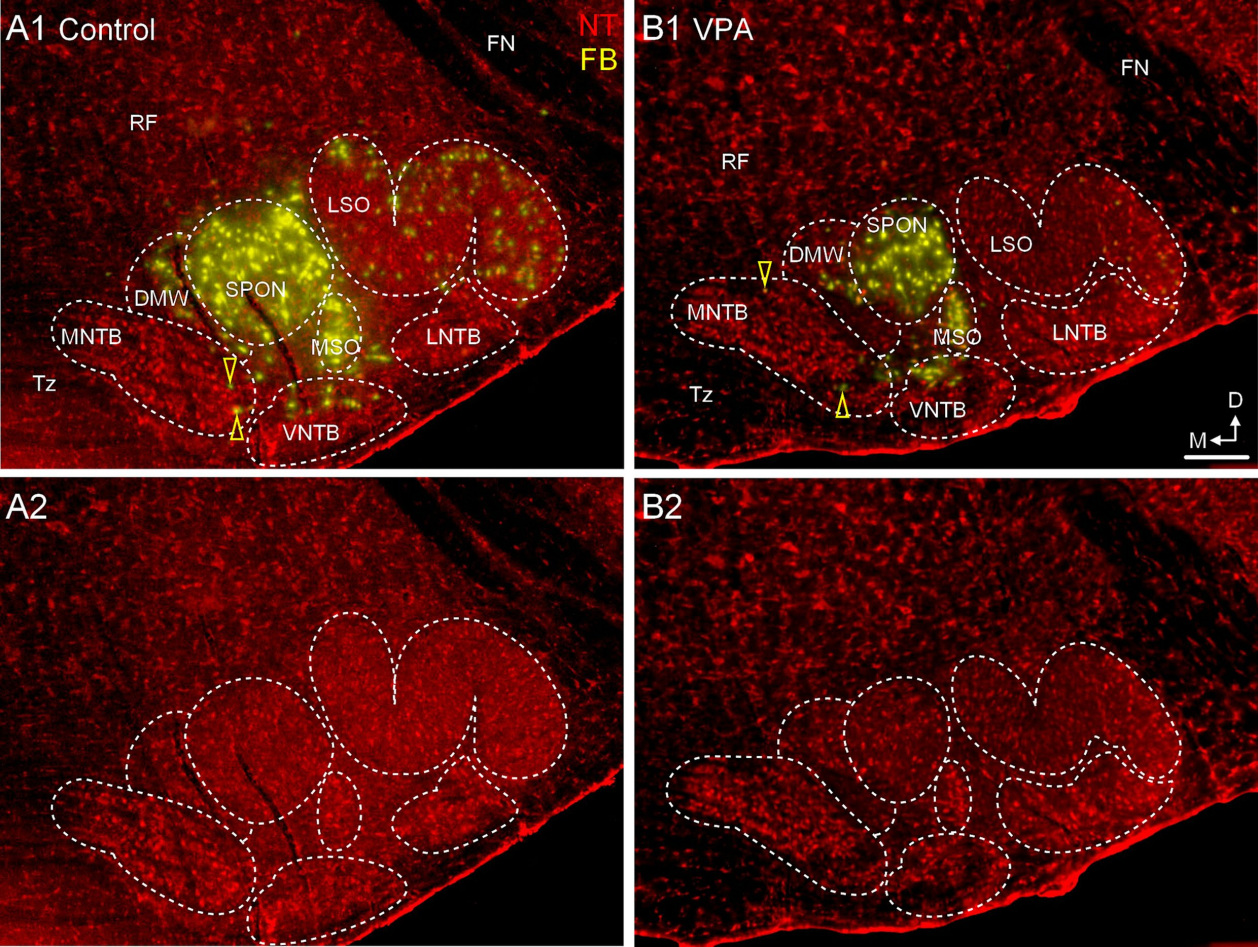
Only occasional MNTB neurons project to the CNIC. Panels **(A1,B1)** show sections through the same rostrocaudal level of the SOC from a control **(A1,A2)** and VPA-exposed animal **(B1,B2)** after tracer injection in the CNIC. Retrogradely-labeled neurons are pseudocolored yellow and Neurotrace counter-stained cells are shown in red (NT). Retrogradely labeled MNTB neurons are indicated with yellow arrowheads. Panels **(A2,B2)** show the Neurotrace counter-stained sections from panels **(A1,B1)** but without the tracer label for reference. The scale bar is equal to 500 μm. D, Dorsal; FN, Facial nerve; LNTB, Lateral nucleus of the trapezoid body; M, Medial; RF, Reticular formation; Tz, Trapezoid body; VNTB, Ventral nucleus of the trapezoid body.

**Figure 6:**
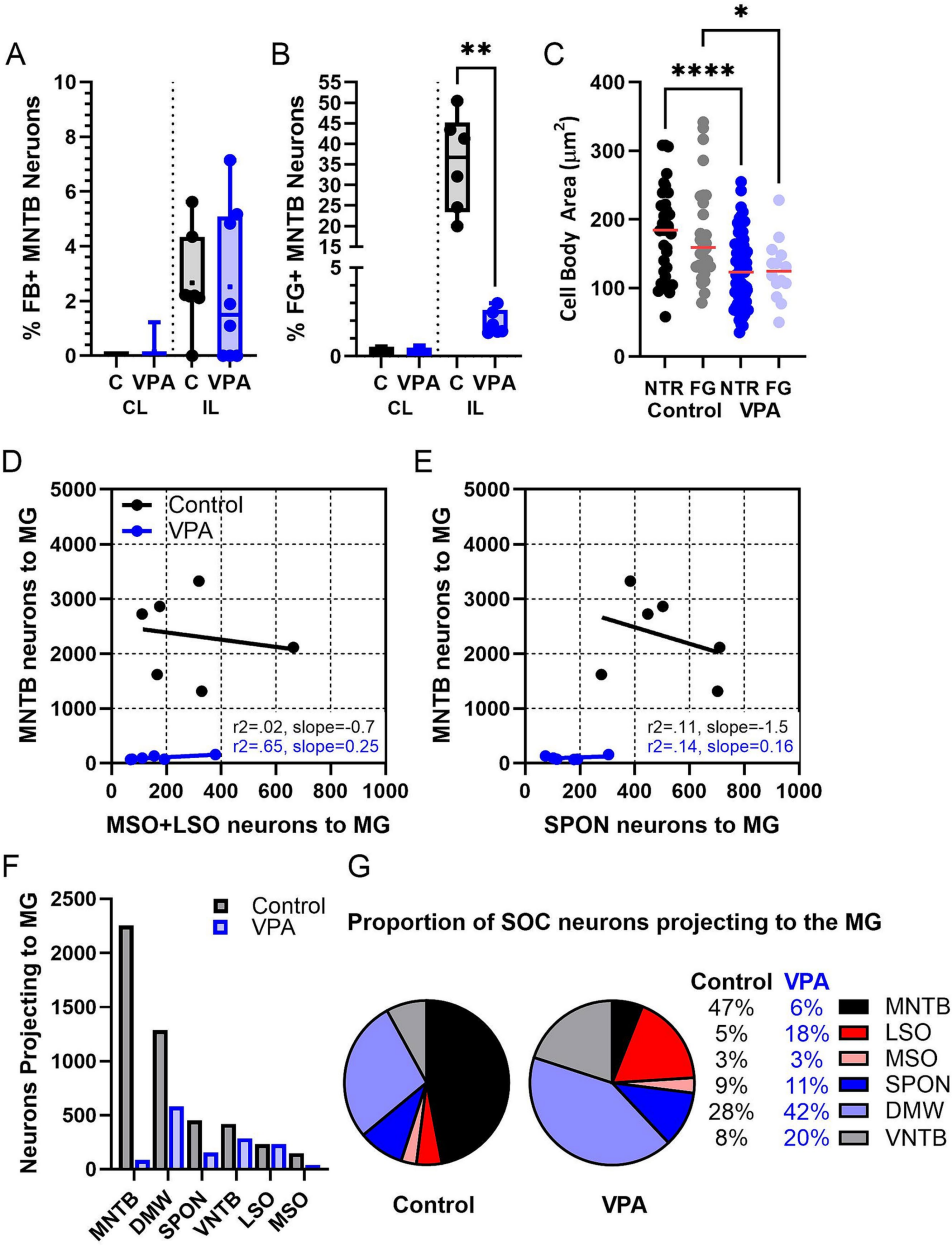
Quantification of the MNTB projection. Panel **(A)** shows the percentage of MNTB neurons that were retrogradely labeled from the CNIC (Mann–Whitney). Panel **(B)** shows the percentage of MNTB neurons retrogradely labeled from the MG (Mann–Whitney). Each data point is averaged data from one animal. Panel **(C)** shows the cross-sectional area of MNTB neurons in control and VPA-exposed animals comparing counterstained (NTR) and retrogradely labeled (FG) neurons (ANOVA). Panel **(D)** shows linear correlation comparing the number of neurons projecting to the MG from the MSO and LSO to the number of neurons projecting to the MG from the MNTB. Panel **(E)** shows linear correlation comparing the number of neurons projecting to the MG from the SPON to the number of neurons projecting to the MG from the MNTB. In Panel **(D,E)**, each data point corresponds to one animal. Panel **(F)** shows the mean number of SOC neurons projecting to the MG in control and VPA-exposed animals. Panel **(G)** shows the distribution of neurons participating in the olivogeniculate projection. The control chart is based on distribution of 7,166 neurons and the VPA chart is based on distribution of 825 neurons. C, Control; CL, Contralateral; DMW, Dorsal medial wedge; FG, Fluorogold; IL, Ipsilateral; LSO, Lateral superior olive; MSO, Medial superior olive; NTR, Neurotrace red; SPON, Superior paraolivary nucleus. Key to symbols: *p < 0.05; **p < 0.01, and ****p < 0.0001.

After injections of FB or FG into the CNIC (Figures 3, 4), only 2.2% (95% CI: 0–5.62%) of neurons in the ipsilateral MNTB were retrogradely labeled in control animals (Figure 5A). In VPA-exposed animals, 1.49% (0–7.14%) of neurons in the ipsilateral MNTB were labeled (Figure 5B). This difference was not significant [U(7,8) = 22.5, *p* = 0.54; Figure 6A]. Contralateral to the injection site, no retrogradely labeled MNTB neurons were found in control or VPA-exposed animals (Figure 5A).

### Projections to the MG

**Figure 7:**
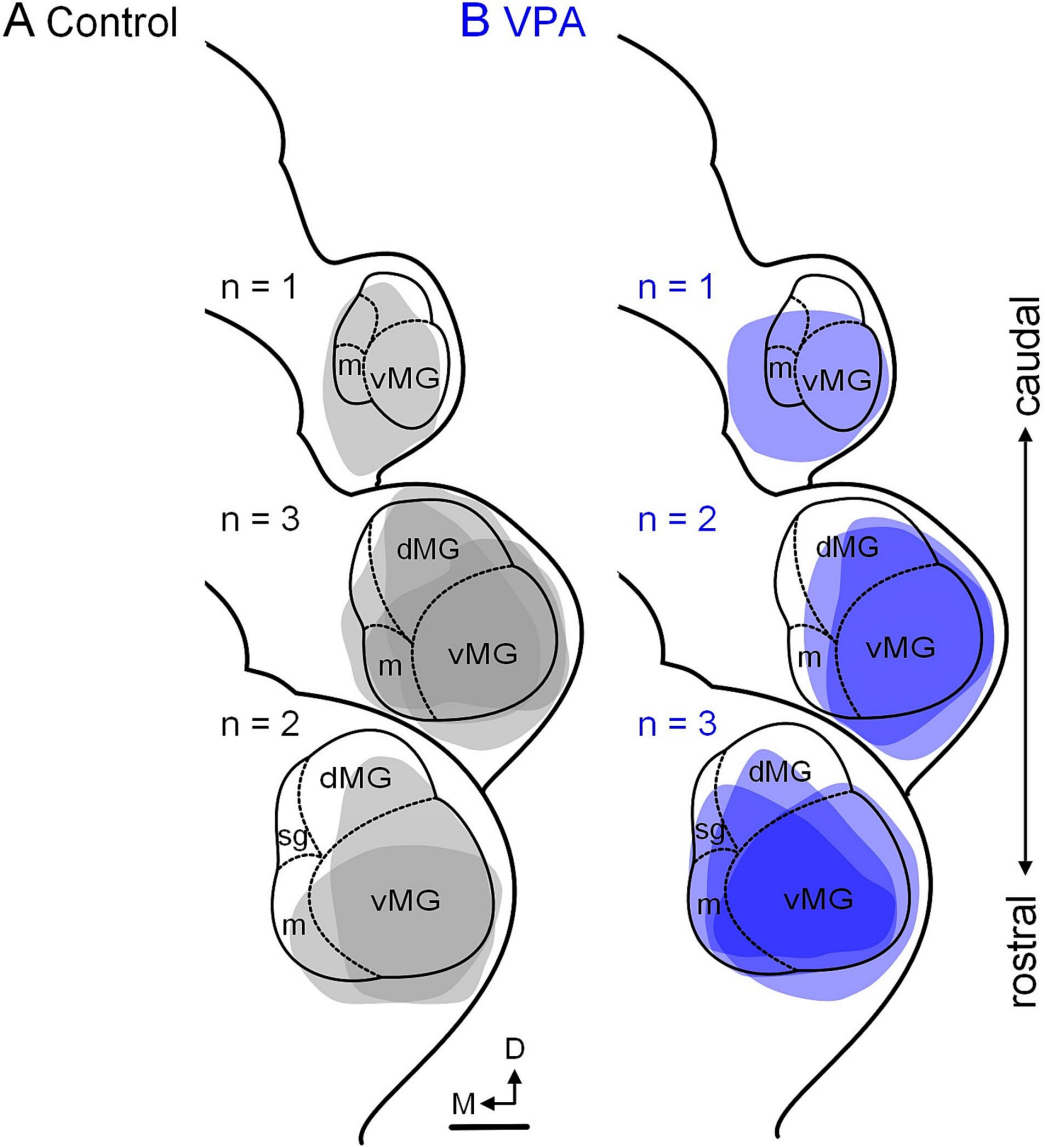
Tracer injection sites in the MG. Injection sites for the six control animals are shown in panel **(A)** and those for the six VPA-exposed animals are shown in panel **(B)**. These cases were selected to match injections site size and rostrocaudal distribution of injections between control and VPA-exposed animals. The scale bar is equal to 200 μm. D, Dorsal; dMG, Dorsal nucleus of the medial geniculate; m, Medial nucleus of the medial geniculate; M, Medial; vMG, Ventral nucleus of the medial geniculate.

**Figure 8:**
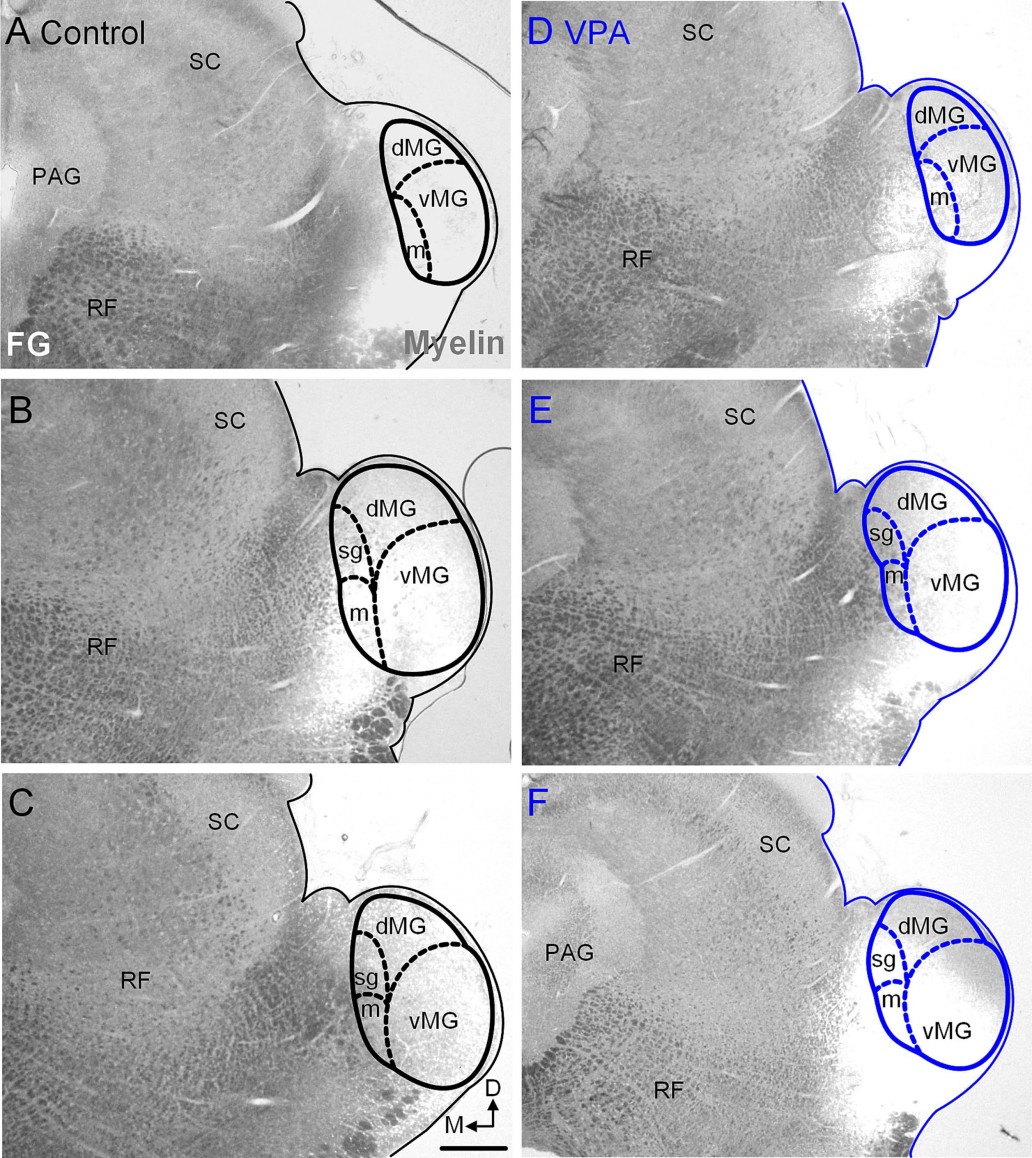
Injection sites in the MG. Representative examples of recovered injections sites in the MG are provided for control **(A–C)** and VPA-exposed animals **(D–F)**. The images were taken with simultaneous illumination with white light and a mercury lamp with a UV filter cube. Myeloarchitecture is shown in gray and the tracer injection in white. The scale bar is equal to 200 μm. D, Dorsal; dMG, Dorsal nucleus of the medial geniculate; m, Medial nucleus of the medial geniculate; M, Medial; vMG, Ventral nucleus of the medial geniculate.

**Figure 9:**
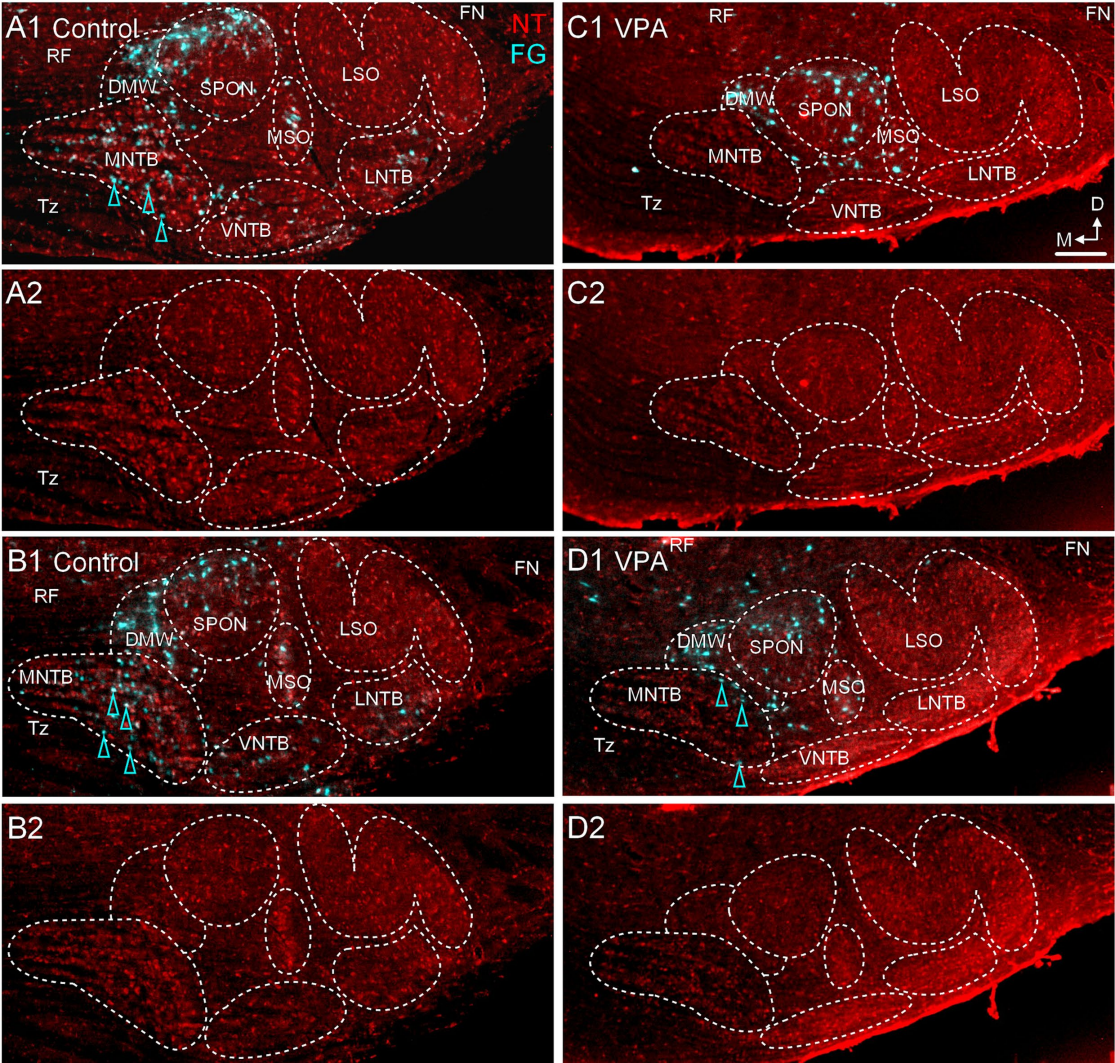
VPA exposure abolishes the projection from the MNTB to the MG. Retrograde labeling from a deposit of FG in the MG is shown for control **(A1, B1)** and VPA-exposed animals **(C1,D1)**. Panels **(A1,A2,B1,B2)** are from two different control animals; panels **(C1,C2,D1,D2)** are from two different VPA-exposed animals. Retrogradely labeled neurons are pseudocolored cyan and Neurotrace counter-stained cells are shown in red (NT). Retrogradely labeled neurons are indicated with cyan arrowheads. Panels **(A2,B2,C2,D2)** show Neurotrace counter-stained sections without the tracer label for reference. The scale bar in panel **(C1)** is equal to 200 μm and applies to all images. D, Dorsal; FN, Facial nerve; LNTB, Lateral nucleus of the trapezoid body; M, Medial; RF, Reticular formation; Tz, Trapezoid body; VNTB, Ventral nucleus of the trapezoid body.

After injections of FG into the MG (Figures 7, 8), 36.7% (20–50%) of neurons in the ipsilateral MNTB were retrogradely labeled in control animals (Figures 9A,B). In VPA-exposed animals, only 1.6% (1.3–2.9%) of neurons in the ipsilateral MNTB were labeled (Figures 9C,D). This difference was significant [U(6,6) = 0, *p* = 0.001; Figure 6B]. Contralateral to the injection site, only 0.43% (0–0.55%) MNTB neurons were retrogradely labeled in control animals and 0.18% (0–0.59%) MNTB neurons were labeled in VPA-exposed animals. This difference was not significant [U(6,6) = 14, *p* = 0.57; Figure 6B]. In control animals, MNTB neurons had a cross-sectional area of 182.5 ± 65.37 μm^2^. MNTB neurons retrogradely labeled from the MG had a cross-sectional area of 177.6 ± 70.23 μm^2^. This difference was not significant (*p* = 0.98; Figure 6C). In VPA-exposed animals, MNTB neurons had a cross-sectional area of 125.1 ± 50.93 μm^2^ and those retrogradely labeled from the MG had a cross sectional area of 126.1 ± 43.88 μm^2^. This difference was not significant (*p* > 0.99; Figure 6C). Consistent with our previous reports, MNTB neurons in control animals are significantly larger than those in VPA-exposed animals [*F*(3, 144) = 10.84, *p* < 0.0001]. Retrogradely, labeled neurons in the MNTB of control animals were significantly larger than those in VPA-exposed animals (*p* = 0.035; Figure 6C).

### Proportion of SOC neurons projecting to the MG

Our previous studies indicate the rat MNTB contains 6,591 neurons in control animals and 5,300 neurons in VPA exposed animals (Table 1, columns A and B; Zimmerman et al., 2018). This equates to a 20% loss of neurons in the MNTB after VPA exposure. Our tract tracing experiments indicate that 36.7% of MNTB neurons in control and only 1.6% of MNTB neurons in VPA-exposed animals project to the ipsilateral MG. Based on these values, we estimate that in control animals 2,419 MNTB neurons project to the ipsilateral MG (Table 1, column F). However, after VPA exposure, this drops to only about 85 total neurons (Table 1, column G) and constitutes a loss of 96% of the MNTB projection to the MG.

In control animals, the vMG and mMG combined include 46,796 neurons, but only 23,403 neurons in VPA-exposed animals (Mansour et al., 2021b; Table 1, columns A and B). This equates to a 50% loss of neurons in the vMG and mMG after VPA exposure. Accordingly, there are nearly 15 times as many MG neurons per MNTB neuron projecting to the MG in VPA-exposed animals (Table 1, column H and I). This drastic change led us to ask if the MNTB projection to the MG was more severely impacted by VPA exposure than other SOC nuclei. Figure 6D shows linear correlation lines comparing the projection to the MG from the MSO and LSO combined and the MNTB alone. In control animals about 320 neurons from the ipsilateral MSO and LSO combined project to the MG while approximately 2,400 MNTB neurons make this projection. In VPA-exposed animals, only about 114 neurons from the MSO and LSO project to the MG, while only about 85 MNTB neurons make this projection (Figure 6F). Figure 5E shows linear correlation lines comparing the projection to the MG from the SPON and the MNTB. In control animals, approximately 450 SPON neurons project to the MG and only about 160 SPON neurons in VPA-exposed animals make this projection (Table 1, columns F and G) (Figure 6E). Together, this suggests that VPA exposure results in a ~ 65% decrease in the projection from the MSO and LSO and a 66% decrease in the projection from the SPON to the MG (Figure 6F). However, VPA exposure results in a 96% decrease in the projection from the MNTB to the MG (Figure 6F; Table 1, column F and G).

Finally, we asked if *in utero* VPA exposure altered the contributions of the SOC nuclei to the olivogeniculate projection (Mansour et al., 2021b). The proportional contributions of each SOC nucleus to the olivogeniculate projection are shown in Figure 5G. The control pie chart is based on distribution of 4,075 neurons; the VPA pie chart is based on distribution of only 840 neurons. The largest projection to the MG is from the medial SOC in control animals: 91% of the olivogeniculate projection comes from the SPON, DMW and MNTB. This proportion changes drastically after VPA exposure. In VPA-exposed animals, the SPON, DMW and MNTB contribute only 75% of the MG projection and the MSO and LSO contribute ~25%. In control animals, the largest single contributor to the olivogeniculate project is the MNTB (53%, Figure 6G). While in VPA-exposed animals the DMW is the largest single contributor (53%) followed by the LSO (21%, Figure 6G).

Since all auditory brainstem nuclei have fewer neurons after VPA exposure (except the VNTB: Mansour and Kulesza, 2021), we calculated a projection change. This calculation compares neuron loss and projection loss (Table 1, column K). A negative value indicates loss of projections beyond what is predicted by VPA-induced neuron loss. The MNTB has a projection change of −11.94 and the VCN has a projection change of −3.59, indicating these are the most severely affected of the brainstem nuclei.

## Discussion

This study provides the first detailed examination of the impact of *in utero* exposure to the antiepileptic VPA on a novel, glycinergic projection from the MNTB to the auditory thalamus. It is important here to recall our finding that VPA exposure results in significantly smaller brains and brainstems (Zimmerman et al., 2018; Mansour et al., 2019). Accordingly, VPA animals received proportionally larger tracer deposits in the CNIC and MG and in theory this should result in more retrogradely labeled neurons, but this was not the case. *In utero* VPA exposure results in hypoplasia and dysmorphology in the auditory brainstem and thalamus, abnormal patterns of CB immunolabeling, reduced ascending projections to the CNIC and MG, abnormal auditory brainstem responses and imbalanced excitatory/inhibitory inputs to brainstem neurons (Zimmerman et al., 2018; Mansour et al., 2019; Zimmerman et al., 2020; Alhelo and Kulesza, 2022). Similar morphological changes have been found in the SOC of human subjects with ASD, including significantly fewer neurons in the MNTB (Kulesza and Mangunay, 2008; Kulesza et al., 2011; Lukose et al., 2015). Therefore, our findings may provide insight into structural and functional changes in the auditory pathway of subjects with ASD and other neurodevelopment disorders.

### Connectivity of the MNTB

The MNTB receives a fast and precise glutamatergic input from GBCs in the contralateral VCN via the calyx of Held (Harrison and Irving, 1964; Morest, 1968a,b; Kuwabara et al., 1991; Smith et al., 1991). Consequently, evoked responses from MNTB principal neurons maintain the precision of the auditory nerve and GBCs. Principal MNTB neurons use glycine as a neurotransmitter (Moore and Caspary, 1983; Wenthold et al., 1987) and project to surrounding nuclei in the ipsilateral SOC (Moore and Caspary, 1983; Kuwabara and Zook, 1992; Sommer et al., 1993; Smith et al., 1998), the ipsilateral VCN (guinea pig: Schofield, 1994), and VNLL and INLL (Spangler et al., 1985; Sommer et al., 1993; Smith et al., 1998; Kelly et al., 2009; Saldaña et al., 2009). Glycinergic input from the MNTB to the MSO and LSO plays essential roles in coding sound source localization (Zarbin et al., 1981; Moore and Caspary, 1983; Kuwabara and Zook, 1992; Grothe and Sanes, 1993; Kapfer et al., 2002). In the SPON, MNTB inputs form temporally precise rebound responses that code the offset of tone-pips and rapid fluctuations in the stimulus envelope (Kulesza et al., 2003; Kadner et al., 2006). The projection from the MNTB to the MG is a recent discovery—it was first described in guinea pigs (Schofield et al., 2014b), but in this species appears to originate from non-principal MNTB neurons. In rats, deposits of retrograde tracers in the vMG and dMG result in robust labeling in the ipsilateral MNTB (Burchell et al., 2022). Our injections in the MG resulted in labeling across the auditory brainstem, consistent with previous reports of thalamic projections from the CNIC, NLL, SOC, and VCN from other species (ferrets: Angelucci et al., 1998; guinea pig: Schofield et al., 2014a,b; rat: Mansour et al., 2021b). It should be emphasized that in control animals, focal injections of FG restricted to the vMG resulted in labeling of up to 84% of MNTB neurons (Burchell et al., 2022). Approximately 87% of MNTB neurons retrogradely labeled from the vMG are CB immunoreactive, confirming that at least in the rat, the thalamic projection from the MNTB is derived from principal neurons (Burchell et al., 2022). Deposits of an anterograde tracer in the MNTB resulted in labeled axons and terminals in the SPON, LSO, VNLL, (consistent with previous reports; see above) but also the ipsilateral nucleus of the brachium of the inferior colliculus and vMG (Burchell et al., 2022). Consistent with a glycinergic projection to the auditory thalamus, there is dense somatic immunolabeling for the glycine receptor in the dMG and vMG, with only scant labeling in the mMG (Burchell et al., 2022). Together, these results support the presence of a prominent projection from MNTB principal neurons in rats providing a fast, glycinergic input to the vMG.

### Functions of the MNTB projection to the auditory thalamus

The role of direct projections from the cochlear nuclei and SOC to the MG are unclear. Specifically, there is a direct projection from stellate neurons in the VCN to the contralateral CNIC and MG (Malmierca et al., 2002; Schofield et al., 2014a; Zimmerman et al., 2020). Our results show that 13,913 neurons in the rat VCN project to the contralateral CNIC (Zimmerman et al., 2020) and 7,164 neurons in the VCN project to the contralateral MG (Mansour et al., 2021b). In the current study, we show that 2,419 MNTB neurons project to the ipsilateral MG. Outside of the VNLL, whose thalamic projection has not yet been examined in rats, the VCN appears to be the largest single subcollicular source of input to the MG, followed by the MNTB (Table 1; Mansour et al., 2021b). The stellate neuron input from the VCN is most likely excitatory based on the nature of VCN projections to the CNIC (Ito and Oliver, 2010) and seems to be most heavily directed to the contralateral mMG (Malmierca et al., 2002; Schofield et al., 2014a). The mMG receives input from the inferior colliculus, CN, SOC, NLL (Schofield et al., 2014a,b; Malmierca et al., 2002; Anderson et al., 2006) and several non-auditory sources. The mMG also receives input from the vestibular system (Roucoux-Hanus and Boisacq-Schepens, 1977) and spinothalamic tract relaying pain and thermal sense (LeDoux et al., 1987). The mMG projects to auditory, somatosensory and prefrontal cortex (Spreafico et al., 1981; Avedafio and Llamas, 1984; Winer, 1985), amygdala (Ottersen and Ben-Ari, 1979; LeDoux et al., 1985; Doron and LeDoux, 1999) and provides descending input to the auditory brainstem (Whitley and Henkel, 1984). Together, these features of the mMG illustrate its possible role in integration across several sensory modalities. The MNTB input is most likely glycinergic and mainly targets the vMG (Burchell et al., 2022). The vMG is the main relay of ascending auditory information from the CNIC to the auditory cortex (Ryugo and Killackey, 1974; LeDoux et al., 1987; Winer and Larue, 1987; González-Hernández et al., 1991; Clerici and Coleman, 1990; Winer et al., 1999; Kimura et al., 2003; Hazama et al., 2004; Ito and Oliver, 2012; Smith et al., 2012) and therefore its functions are likely focused on hearing. The roles these inputs from the VCN and MNTB play in shaping responses of neurons across the MG subdivision are unclear, but they clearly provide fast and precise input to the auditory thalamus (Schofield et al., 2014b). It is important to emphasize that based on tract tracing studies in rat and guinea pig (Malmierca et al., 2002; Schofield et al., 2014a,b; Burchell et al., 2022), the VCN and MNTB projections are targeting different regions of the MG and would appear to be functionally independent. As such, we will only discuss the MNTB projection further.

Within the SOC, glycinergic input from the MNTB to the MSO arrives before excitatory, glutamatergic inputs from the ipsilateral VCN, despite a longer axon distance and extra synapse (Grothe, 1994; Grothe and Sanes, 1993; Roberts et al., 2014). In the rat, MNTB principal neurons have spontaneous discharge rates of 20–30 spikes/s and respond to pure tone-pips with precise temporal patterns of action potentials; shortly following the stimulus offset, MNTB neurons have a brief window of quiescence and then gradually resume their spontaneous discharge rate (Kulesza et al., 2003; Kulesza, 2007; Kadner et al., 2006; Kadner and Berrebi, 2008; Kopp-Scheinpflug et al., 2008). This post-stimulus interruption in MNTB responses to pure tone-pips is essential in the formation of offset responses in the SPON (Kulesza et al., 2003; Kadner et al., 2006). Together, these findings suggest that glycinergic input from the MNTB likely reaches the vMG before any other lemniscal inputs, but more importantly provides fast and temporally precise inhibition. Additionally, based on our tracer injections in the IC and MG, it appears that the MNTB projection to the vMG has very few if any collaterals to the CNIC (Figure 8)—this is unique among nuclei in the CN, SOC and NLL projecting to the thalamus. The reason for this projection pattern is unclear but we propose that maintenance of the timing and integration of this glycinergic input is functionally important for at least a subset of functionally distinct vMG neurons (see below).

*In vivo* recordings in rabbits reveal that about 8% of neurons in the rabbit vMG respond to pure tones with offset responses and about 3% of neurons respond to pure tone-pips with on/off responses (Cetas et al., 2002). Similar offset-type responses have been found in and around the vMG of other species (guinea pig: Yu et al., 2004; cat: Aitkin and Prain, 1974, mouse: Anderson and Linden, 2016). Our immunolabeling for the glycine receptors in the dMG and vMG is largely somatic, similar to what is found in the SPON (Kulesza and Berrebi, 2000), where glycinergic inputs play an essential role in forming offset responses (Kulesza, 2007; Burchell et al., 2022). Based on the role of the MNTB in forming offset responses in the SPON and the distribution of glycine receptor positive puncta in the vMG, we hypothesize that the MNTB input to this region functions, at least in part, to create responses timed to the stimulus offset.

### Impact of VPA exposure on connectivity in the auditory brainstem

In control animals, we estimate about 20,326 total neurons in the SOC (LSO, MSO, MNTB, SPON, VNTB, LNTB and DMW). However, in VPA-exposed animals, there are only 15,136 total neurons in these nuclei—this equates to a 26% decrease in the total number of neurons in the SOC. However, VPA exposure results in approximately a 49% decrease in the SOC projection to the CNIC (Zimmerman et al., 2020) and a 73% decrease in the SOC projection to the MG (Mansour et al., 2021b; Mansour and Kulesza, 2021). Additionally, VPA exposure appears to result in a significant reorganization of the olivogeniculate projection in rats. VPA exposure results in a 20% decrease in the total number of MNTB neurons (Zimmerman et al., 2018) but a 96% decrease in the total number of MNTB neurons projecting to the MG (Mansour et al., 2021b). VPA exposure results in a 25% decrease in the total number of LSO neurons (Zimmerman et al., 2018). The ascending projection from the LSO to the ipsilateral CNIC is inhibitory and likely glycinergic, while the projection to the contralateral CNIC is glutamatergic (Ito and Oliver, 2010). VPA exposure results in a 34% decrease in the ipsilateral projection and a 47% decrease in the contralateral projection from the LSO to the CNIC (Zimmerman et al., 2020). However, VPA exposure resulted in a 57% decrease in the ipsilateral projection (glycine) but a 172% increase in the contralateral projection to the MG from the LSO (Mansour et al., 2021b). The MSO projection to the MG is likely glutamatergic. VPA exposure results in a 57% decrease in the total number of MSO neurons (Zimmerman et al., 2018), a 74% decrease in the number of MSO neurons projecting to the CNIC (Zimmerman et al., 2020) and 74% decrease in the number of neurons projecting to the MG (Mansour et al., 2021b). The SPON projection to the MG is most likely GABAergic (Kulesza and Berrebi, 2000). VPA exposure results in a 43% decrease in the total number of SPON neurons (Zimmerman et al., 2018), a 61% decrease in the number of SPON neurons projecting to the CNIC and a 66% decrease in the total number of SPON neurons projecting to the MG (Mansour et al., 2021b). While VPA exposure results in significant reorganization of the olivogeniculate projection (Figure 5G), there is still a net reduction in the number of SOC neurons projecting to the thalamus. Specifically, the proportion of VNTB and DMW neurons projecting to the MG increases (Figure 5G), but there is an overall reduction in the number of neurons projecting to the MG from these nuclei (Table 1, column K). The only exception to this pattern is from the contralateral LSO (Table 1, column K). There are glycinergic neurons in the rat LSO, VNTB and LNTB (Wenthold et al., 1987; Rampon et al., 1996) and changes in projections from these nuclei may compensate for the loss of glycinergic input from the MNTB, although they likely cannot provide the same temporal precision. Besides the MNTB, these results do not currently provide a clear pattern for the impact of *in utero* VPA exposure on projections of specific nuclei or neurotransmitter systems in the auditory brainstem. VPA has been shown to inhibit neurite outgrowth (Qian et al., 2009), and we propose that the drastic reduction in the thalamic projection of the MNTB is due to the impact of VPA on developing axons. Nonetheless, these results emphasize two important points. First, our findings are consistent with loss of projections to both the CNIC and MG beyond what is predicted from neuron loss alone (Table 1, column K). Second, VPA exposure seems to have a preferential impact on longer axonal projections and the MNTB projection to the thalamus in particular.

As we have previously shown, the number of neurons from the VCN and most SOC nuclei projecting to the MG are fewer than those projecting to the CNIC. There are however two exceptions to this: the DMW and MNTB (Burchell et al., 2022). In control animals, we estimate that only about 810 DMW neurons (combined ipsilateral and contralateral) project to the CNIC but 1,286 neurons project to the vMG (1.6-fold larger thalamic projection). In VPA-exposed animals we estimate that 174 DMW neurons project to the CNIC and about 583 neurons project to the vMG (3.3-fold larger thalamic projection). This is mainly attributable to a nearly 10-fold decrease in the projection from the ipsilateral DMW to the vMG after VPA exposure. In control animals, we estimate that only about 145 MNTB neurons project to the CNIC but 2,256 neurons project to the vMG (16-fold larger thalamic projection). In VPA-exposed animals we estimate that approximately 80 MNTB neurons project to the CNIC and only about 85 neurons project to the vMG. This difference in the number of MNTB neurons projecting to the MG compared to the CNIC in VPA-exposed animals is essentially 0 and is consistent with abolishment of the thalamic projection from the MNTB (Figure 1). This finding is based on injections of the retrograde tracer FG into the MG. Anterograde tracing experiments will undoubtably provide additional insight into course, collaterals and distribution of MNTB axons beyond the SOC. It is not clear to what degree MNTB projections to the CN, SPON, MSO, LSO and NLL are impacted by *in utero* VPA exposure, but we will explore these projections in future studies.

### Impact of loss of MNTB input to the vMG

The role of glycinergic input to the VNLL, INLL or vMG from the MNTB is not well characterized, so we can only speculate on the impact of losing this projection. Again, we propose that at least in the vMG, the MNTB input contributes to formation of offset responses. VPA exposure appears to abolish this projection to the vMG and so we hypothesize that VPA-exposed animals have significantly fewer offset responding neurons in the vMG. While there are significantly fewer MNTB and SPON neurons in VPA-exposed animals, we have not examined the distribution of glycine receptors or glycine-immunoreactive puncta in the SPON. MNTB principal neurons are characteristically CB+, but VPA exposure results in reduced CB immunolabeling in the MNTB, and many MNTB neurons have CB immunoreactivity restricted to the nucleus (Zimmerman et al., 2018), so counting CB+ puncta may not be a viable metric to quantify this projection to the SPON, VNLL or INLL. Regardless, VPA exposure results in near complete loss of MNTB input to the vMG. Since the MNTB input would provide a fast, glycinergic input to vMG neurons we hypothesize that VPA-exposed animals have reduced coding of temporal information in the auditory thalamus. This most likely impairs coding of complex sounds such as vocalizations.

Interestingly, the physiological impact of abolishing the thalamic projection from the MNTB may not be so clear cut. Mice with deletion of the transcription factor *En1*, lack MNTB and VNTB neurons but neurons in the LSO and SPON still receive glycinergic innervation (Jalabi et al., 2013; Altieri et al., 2014). Mice lacking an MNTB had normal sound-evoked startle responses, but elevated thresholds to pure tones, significantly reduced amplitude of wave III, which is attributed largely to the MNTB and reduced sound localization abilities (Jalabi et al., 2013). However, these mice have fewer GlyT2+ puncta in the LSO but no change in the SPON. The development of these GlyT2+ puncta in the LSO even followed a similar time course as control animals, but was delayed in the SPON (Altieri et al., 2014). In fact, strychnine-sensitive offset responses could still be elicited in the SPON, despite there being no MNTB neurons (Jalabi et al., 2013). The origin of these glycinergic inputs to the SPON and LSO in the absence of MNTB neurons has not been resolved, but likely arise from the contralateral VCN (Jalabi et al., 2013).

Our tract tracing studies show that the projection to the MG from the contralateral VCN is greatly reduced in VPA-exposed animals (Table 1). Specifically, deposits of retrograde tracers in the MG results in labeling of about 7,166 VCN neurons in control animals but only 825 neurons in VPA-exposed animals (Mansour et al., 2021b)—this equates to a 88% decrease. While the VCN may reprogram to compensate for loss of local glycinergic projections in *En1* deficit animals, this does not appear likely for long-range projections in VPA-exposed animals. Therefore, we hypothesize significant loss of glycinergic innervation of the vMG and dMG in VPA-exposed animals. Regardless, our results are consistent with significantly reduced and disproportionate ascending thalamic projections in VPA-exposed animals. These changes likely translate into impaired temporal and spectral coding of auditory information in the MG and auditory cortex and provide evidence that certain projections may be preferentially impacted in animal models of ASD.

## Data Availability

The raw data supporting the conclusions of this article will be made available by the authors, without undue reservation.
